# Antibiofilm Effect of DNase against Single and Mixed Species Biofilm

**DOI:** 10.3390/foods7030042

**Published:** 2018-03-19

**Authors:** Komal Sharma, Ankita Pagedar Singh

**Affiliations:** 1 Ashok & Rita Patel Institute of Integrated Study and Research in Biotechnology and Allied Sciences, New Vallabh Vidya Nagar, Anand 388121, Gujarat, India; komalsharma0325@gmail.com; 2 Department of Food Processing Technology, AD Patel Institute of Technology, New Vallabh Vidya Nagar, Anand 388121, Gujarat, India

**Keywords:** biofilms, DNase I, pre-treatment, post-treatment, mixed species biofilm, disintegration of matrix, antibiofilm methods

## Abstract

Biofilms are aggregates of microorganisms that coexist in socially coordinated micro-niche in a self-produced polymeric matrix on pre-conditioned surfaces. The biofilm matrix reduces the efficacy of antibiofilm strategies. DNase degrades the extracellular DNA (e-DNA) present in the matrix, rendering the matrix weak and susceptible to antimicrobials. In the current study, the effect of DNase I was evaluated during biofilm formation (pre-treatment), on preformed biofilms (post-treatment) and both (dual treatment). The DNase I pre-treatment was optimized for *P. aeruginosa* PAO1 (model biofilm organism) at 10 µg/mL and post-treatment at 10 µg/mL with 15 min of contact duration. Inclusion of Mg^2+^ alongside DNase I post-treatment resulted in 90% reduction in biofilm within only 5 min of contact time (irrespective of age of biofilm). On extension of these findings, DNase I was found to be less effective against mixed species biofilm than individual biofilms. DNase I can be used as potent antibiofilm agent and with further optimization can be effectively used for biofilm prevention and reduction in situ.

## 1. Introduction

Microorganisms prefer to coexist in an extremely coordinated surface adhered lifestyle, known as biofilm. Biofilms are a grave concern across various industries like food, textile, paper, oil, aviation, shipping and even the medical sector. They have accounted for reduced efficacy of heat exchange processes, corrosion of materials, blocking of membranes and degradation of ship hulls. Biofilms mediated infections contribute to almost 80% of clinical infections reported globally [1]. In the food industry, biofilms on food contact surfaces pose a food safety hazard and product quality issues. Antibiofilm strategies in the healthcare sector include use of antibiotics and/or biocides. However, in food industry scrapping, hot water treatment, acid/alkali treatments and biocides as a part of the cleaning regime are used to combat biofilms. As biofilms are notorious for being resistant to conventional antibiofilm approaches, alternative antibiofilm strategies like using proteases, amylases, bis-(3′-5′)-cyclic dimeric guanosine monophosphate (c-di-GMP) and quorum sensing inhibitors have been explored [2]. These methods are reportedly more effective for prevention of biofilm formation and may or may not be effective on pre-formed biofilms [3,4,5]. 

For development of an effective antibiofilm strategy, a thorough understanding of the biofilm formation process (initial adhesion, maturation, quorum sensing and dissemination), a metabolic state of biofilm inhabitants and composition of biofilm matrix is required. Biofilm inhabitants display a reduced metabolic rate, enhanced efflux, adaptive and cross-resistances and hence are more resistant to antimicrobials than their planktonic counterparts [6,7]. In addition, the biofilm matrix acts as a protective barrier and reduces the percolation of antimicrobials to a deeper strata of biofilm structure. The biofilm matrix is composed of 40–95% polysaccharides, 1–60% proteins, 1–40% lipids and 1–10% nucleic acid [8]. Prevalence of the e-DNA in the biofilm matrix has been reported in biofilms of several microorganisms, both Gram-positive and Gram-negative. The release of e-DNA is mediated by autolysis (programmed cell death i.e., suicide and altruistic cell death-fratricide) [9] and through vacuoles and membrane vesicles [10]. The e-DNA contributes to cell-surface and cell–cell interactions, horizontal gene transfer, integrity, cohesivity and viscoelasticity of the biofilm matrix and thus plays a major role in biofilm stability [11].

In view of significance of e-DNA in biofilm matrix and biofilm formation [11], it is indeed a potential target for development of antibiofilm strategies by using DNA degrading enzymes i.e., DNase. Antibiofilm effect of DNase has been studied for organisms such as *S. aureus* and *P. aeruginosa*, *E. coli*, *Acinetobacter baumannii*, *Haemophilus influenzae* and *K. pneumoniae*. Most of these studies have been conducted using commercially available DNases like DNase I (derived from bovine pancreas), DNase 1L2 (human keratinocyte DNase), Dornase alpha (recombinant human DNase), λ exonuclease (viral DNase), NucB and streptodornase produced by *Bacillus licheniformis* and *Streptococcus* spp., respectively [12]. Moreover, microorganisms producing nucleases have been shown to form lesser biofilm than their non-nuclease producing mutants [13]. Addition of l-methionine that induces DNase secretion by *P. aeruginosa*, resulted in reduced biofilm formation [14]. The antibiofilm effect of DNase has been studied with or without antibiotics, dispersinB [15,16] and glutathione [17]. Previous studies have reported that, in the presence of DNase, a lower concentration of antibiotics was required to inhibit biofilm formation by *Campylobacter jejuni* [18,19]. Most of the published studies have used DNase in growth medium itself i.e., its addition at time point 0 of biofilm formation. In the current investigation, such a biofilm preventive effect has been described using the term “pre-treatment”.

On the other hand, DNase based treatments of pre-formed biofilms have not been explored much. There are only a few reports available [20,21], which describe application of DNase in combination with proteinase K [22,23], EDTA [24] and dextranase [25] on already formed biofilms. Such a biofilm control/therapeutic effect has been discussed using the term “Post-treatment” in this study. Most of these studies have been carried out on single species biofilm and thus mixed species biofilms yet remain to be explored. Moreover, the antibiofilm effect of DNase evaluated on a single organism may not be directly applicable on in situ biofilms that are formed by mixed species consortia. In purview of reviewed literature, it appears that a study comparing the effect of DNase on biofilms formed by different pathogens will be an addition to the existing knowledge. Therefore, in the current investigation, the antibiofilm efficacy of DNase I treatments (pre and post) were optimized on *P. aeruginosa* PAO1 biofilms. *Pseudomonas* spp. is a concern in food industry due to its inherent antimicrobial resistance and potential to produce heat stable proteases and lipases [26]. Though *P. aeruginosa* is not a typical food related pathogenic organism, its presence in drinking water poses a health hazard [27]. In addition, *P. aeruginosa* forms copious biofilm and thus is considered as a model organism for biofilm formation. In this study, the DNase I treatments optimized using *P. aeruginosa* PAO1 were extended to mixed-species biofilm of organisms (*Staphylococcus aureus*, *Klebsiella* spp., *Enterococcus faecalis*, *Salmonella* Typhimurium) that are relevant to food industry. 

## 2. Materials and Methods 

### 2.1. Culture Maintenance

Microorganisms used in the current investigation were *Pseudomonas aeruginosa* PAO1 (MTCC 3541), *Enterococcus faecalis* (ATCC 29212), *Salmonella* Typhimurium (ATCC 23564) and *Staphylococcus aureus* (ATCC 25923). These cultures were obtained either from the Microbial Type Culture Collection (MTCC) at the Institute of Microbial Technology, Chandigarh, India or American Type Culture Collection (ATCC), Manassas, VA, USA. One *Klebsiella* spp. that was isolated from a biofilm sample obtained from the food industry was also used in this study. The cultures were maintained in tryptone soy broth (TSB) or on agar plates and stored as glycerol stocks at −40 °C. Culture inoculum for experiments was prepared by adjusting optical density (OD) of overnight activated culture to 0.5 (c.a., 8 Log cfu/mL) at 620 nm. All materials and reagents were procured from HiMedia Labs, Mumbai, India, unless specified otherwise. 

### 2.2. Biofilm Formation Assay

The assay was carried out in accordance with a previously published protocol [28]. Briefly, 200 µL of TSB per well of sterile 96-well plate made of polystyrene (Axiva Biotech, New Delhi, India) was inoculated with 20 µL of inoculum and the biofilm was allowed to develop at 37 °C/24 h. Later, the contents of the wells were decanted and wells were washed 3–4 times with sterile PBS (Phosphate Buffered Saline) to dislodge the loosely adhered cells. The remaining biofilms were vigorously blotted on stack of paper towels and air dried [29]. The biofilms were stained with 1% crystal violet, rinsed 3–4 times with water in a large Petri dish to remove the excess stain, blotted on stack of paper towels and air-dried. The crystal violet bound to biofilms was then resolubilized using 33% glacial acetic acid and absorbance was measured at 595 nm (A595 nm; plotted on the primary *y*-axis) using a microplate reader (EPOCH 2c, BIOTEK, Winooski, VT, USA). As a negative control, uninoculated wells containing TSB were treated similarly and readings obtained were subtracted from the test readings. 

### 2.3. Optimization of DNase I Concentration for Pre-Treatment

A gradient of DNase I in the range of 0–50 µg/mL was prepared by dissolving lyophilized powder in nuclease free water and diluting it with 0.15 M NaCl solution to achieve the desired concentrations. For optimization of DNase I concentration for pre-treatment, biofilms of *P. aeruginosa* PAO1 were formed in the presence of different concentrations of DNase I for 24, 48, 72, 96 h and quantified as A595 nm. As negative control, uninoculated wells containing TSB and diluent were treated similarly and readings obtained were subtracted from the test readings. Positive control wells containing inoculated TSB without DNase I were considered as “Control A595 nm”. The biofilm percentage reduction (BPR was calculated as below and plotted on the secondary *y*-axis:

$$\mathrm{BPR}=\left(\frac{\mathrm{Control}\;A595\;\mathrm{nm}-\mathrm{test}\;A595\;\mathrm{nm}}{\mathrm{Control}\;A595\;\mathrm{nm}}\right)\times 100$$

### 2.4. Optimization of Contact Time and Concentration of DNase I for Post Treatment

As described in previous sections, biofilms of *P. aeruginosa* PAO1 were formed for 24, 48, 72, 96 h. The contents of the plate were decanted and rinsed using sterile PBS. The wells containing pre-formed biofilms were refilled with TSB containing DNase I at a concentration optimized for pre-treatment. The plate was left undisturbed to maintain contact duration of 0, 5, 10, 15, 20, 25, 30, 35, 60, 75 and 120 min. Subsequently, the plate was decanted, rinsed, air-dried, stained, destained and quantified as A595 nm. Test controls containing only diluents (0.15 M NaCl) were also evaluated for antibiofilm effects, if any, for respective contact times.

Furthermore, the concentration of DNase I was also optimized in the presence of Mg^2+^ ions (10 mM) for a contact time of 15 min. The antibiofilm effect of Mg^2+^, if any, was also evaluated by setting up a test control containing only Mg^2+^ in absence of DNase I.

### 2.5. Pre-Treatment, Post-Treatment and Dual Treatment of Microbial Biofilms by DNase I

Individual biofilm formation by *P. aeruginosa* PAO1, *E. faecalis*, *S.* Typhimurium, *S. aureus* and *Klebsiella* spp. was done in TSB for 24 h and subjected to pre-treatment, post-treatment and dual treatment using DNase I (without Mg^2+^) as described earlier. Biofilm quantification was done in terms of A595 nm. 

### 2.6. Pre-Treatment, Post-Treatment and Dual Treatment of Mixed Species Biofilm by DNase I

In order to prepare mixed species consortium, either of the pathogen was added in 2× concentration than others in a cocktail (for example: in *P. aeruginosa* PAO1 2× cocktail, the ratio of test pathogens *P. aeruginosa* PAO1: *S. aureus*: *Salmonella* Typhimurium: *E. faecalis*: *Klebsiella* spp. was 2:1:1:1:1). Similarly, 2× cocktails with one of the pathogens as dominant were also prepared, namely, *S. aureus* 2×, *Salmonella* 2×, *E. faecalis* 2×, *Klebsiella* spp. 2× and used for biofilm formation for 24 and 48 h. The biofilms were subjected to DNase I pre-treatment, post-treatment and dual treatment (without Mg^2+^) and quantified as described in previous sections.

### 2.7. Statistical Analysis

All the experiments were conducted in triplicate and minimum three trials were carried out for each experiment. The results were calculated as average values of three readings along with standard deviation depicted as error bars. The average, standard deviation, for the readings obtained was determined by using Microsoft Excel Software (Microsoft Office 2010, Redmond, WA, USA). Statistical tool XL-statistics v4.5 was used for carrying out Student’s *t*-test (with Bonferroni post hoc analysis) and Analysis of variance (ANOVA). The statistical tool is a freeware of set of workbooks for Microsoft excel and available online [29]. ‘Significance’ is expressed at the 5% level (*p* < 0.05) or mentioned otherwise.

## 3. Results

### 3.1. Optimization of Pre-Treatment and Post-Treatment

#### 3.1.1. DNase I Concentration for Pre-Treatment

**Figure 1:**
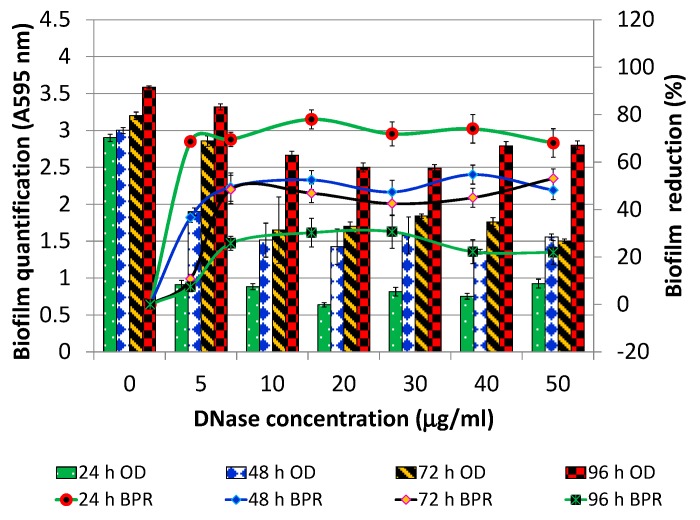
Effect of DNase I (without Mg^2+^) pretreatment on *P. aeruginosa* PAO1 biofilm grown for 24, 48, 72, 96 h in varying concentrations of DNase I (0–50 µg/mL). Biofilm quantification (A595 nm) on the primary *y*-axis and biofilm percentage reduction (line graph) on the secondary *y*-axis. OD: optical density; BPR: biofilm percentage reduction.

*P. aeruginosa* PAO1 biofilm was developed in varying concentrations of DNase I (0–50 µg/mL; without Mg^2+^) for 24, 48, 72, and 96 h. The results are expressed in terms of both biofilm quantification (A595 nm; Figure 1) and biofilm percentage reduction (BPR) (Figure 1). In comparison to control biofilm (DNase concentration 0 µg/mL), a reduction of 68.6% was observed when biofilm was grown for 24 h in the presence of 5 µg/mL of DNase I. The BPR observed for biofilms cultivated for 48, 72, 96 h in 5 µg/mL of DNase I was only 36%, 10%, 7%, respectively. On the other hand, when biofilms were cultivated in the presence of 10 µg/mL DNase I, BPR was found to be 70%, 50%, 48%, 26% for 24, 48, 72, 96 h old biofilms, respectively. Further increase in concentration of DNase I, beyond 10 µg/mL, did not result in significant difference in BPR (*p* > 0.05). The susceptibility of 96 h biofilm was least at all the DNase I concentrations tested. However, the susceptibility of biofilms when cultivated for 48 and 72 h was almost at par (50% biofilm reduction). Based on these findings, 10 µg/mL of DNase I was selected as the optimal concentration for pre-treatment.

#### 3.1.2. DNase I Contact Time for Post-Treatment

**Figure 2:**
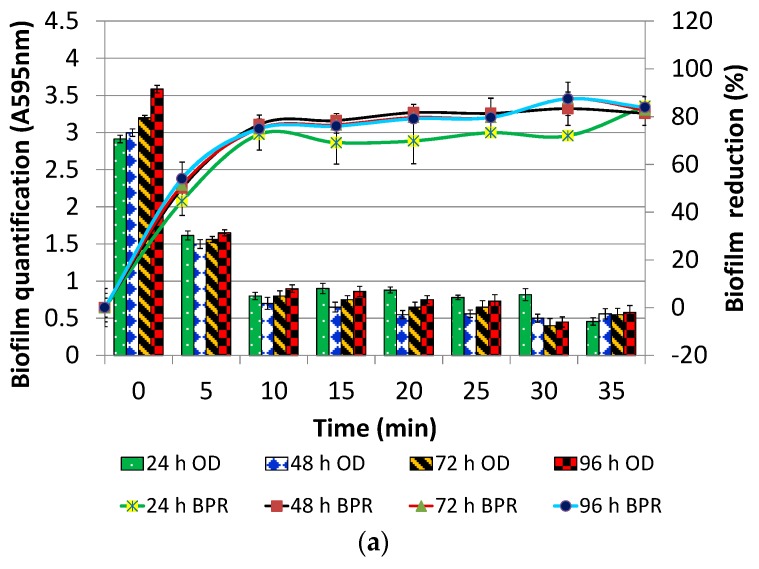

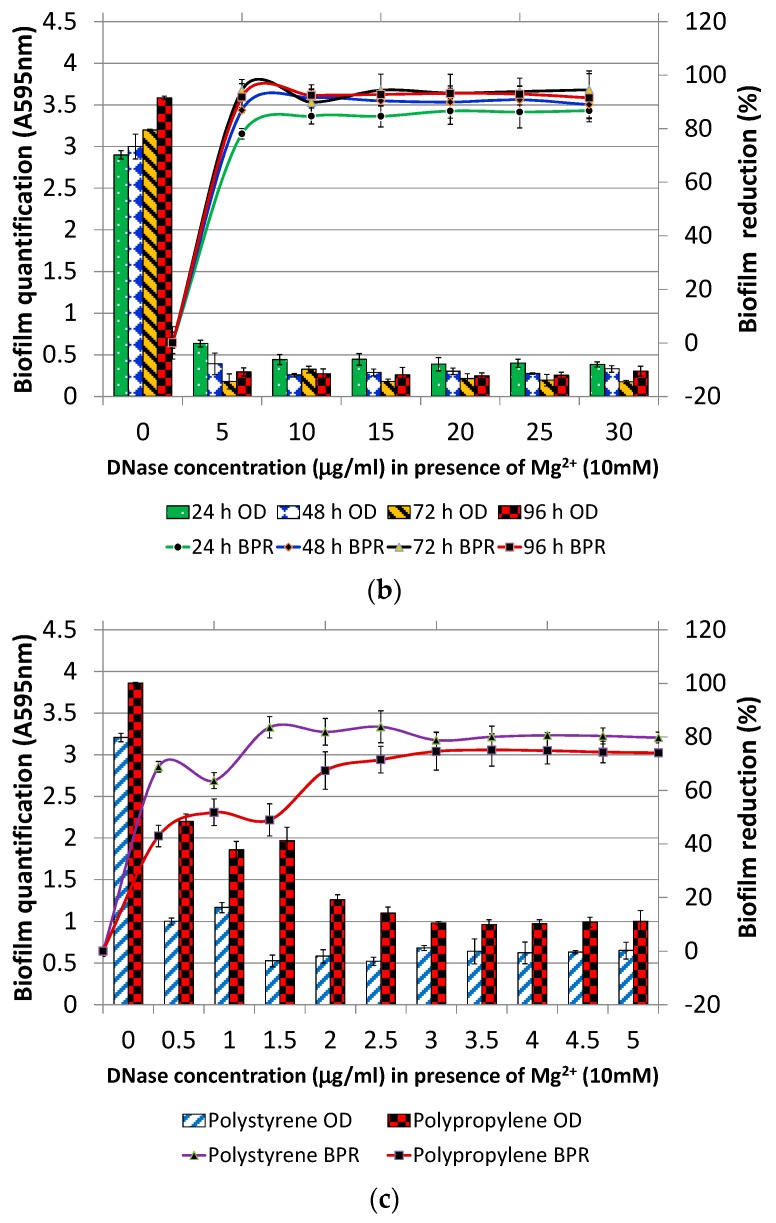
Effect of variable time of post-treatment on 24, 48, 72, 96 h old *P. aeruginosa* PAO1 biofilm with (**a**) DNase (10 µg/mL; without Mg^2+^) and for varying contact time; (**b**) DNase (10 µg/mL) in the presence of Mg^2+^ (10 mM) and for contact time of 15 min; (**c**) variable DNase (µg/mL) in the presence of Mg^2+^ (10 mM) and for contact time of 15 min.

The preformed biofilms of *P. aeruginosa* PAO1 biofilm were treated with 10 µg/mL of DNase I (without Mg^2+^) for varying contact duration ranging from 0 to 120 min. However, the result as presented in Figure 2a has been shown only until 35 min of contact duration as the observations at other contact durations were more or less similar. Irrespective of the age of biofilm, BPR observed was in the range of 45–53% at contact duration of 5 min and 73–77% for contact duration of 10 min of DNase I treatment. Notably, insignificant difference, irrespective of the age of the biofilms, was observed in BPR when post-treatment was done for more than 10 min (*p* > 0.05; Figure 2a). Thus, to be on the safer side, 15 min of contact duration was selected for post-treatment.

Further efficacy of DNase I for post treatment was evaluated in the presence of Mg^2+^ ions (10 mM). It was found that, in the presence of Mg^2+^, DNase I could effectively reduce *P. aeruginosa* PAO1 biofilm by 90% at a concentration of 5 µg/mL irrespective of the age of biofilm (Figure 2b). Increasing the concentration of DNase I in the presence of Mg^2+^ did not result in significant difference in biofilm reduction (*p* > 0.05). A control containing only Mg^2+^ (without DNase I) did not exert any biofilm reduction effect. 

In view of these observations, DNase I concentration in the presence of Mg^2+^ was optimized over a range of 0 to 5 µg/mL in steps of 0.5 while keeping the contact duration constant at 15 min. In this experiment, in addition to polystyrene, antibiofilm efficacy of DNase I was also evaluated on polypropylene. It was found that 1.5 µg/mL and 2 µg/mL of DNase I could effectively reduce the 24 h old *P. aeruginosa* PAO1 biofilm by 80% on polystyrene and 75% on polypropylene, respectively (Figure 2c). The same assay was reconducted at constant DNase I concentration (1.5 µg/mL for Polystyrene and 2 µg/mL for polypropylene), but, for variable contact time, showed that aforementioned antibiofilm efficacy could be achieved within only 5 min of contact duration. It is apparent from the above-mentioned results that Mg^2+^ ions are essential for antibiofilm efficacy of DNase I.

### 3.2. DNase I Treatment (Pre, Post and Dual) of Individual and Mixed Species Biofilms

**Figure 3:**
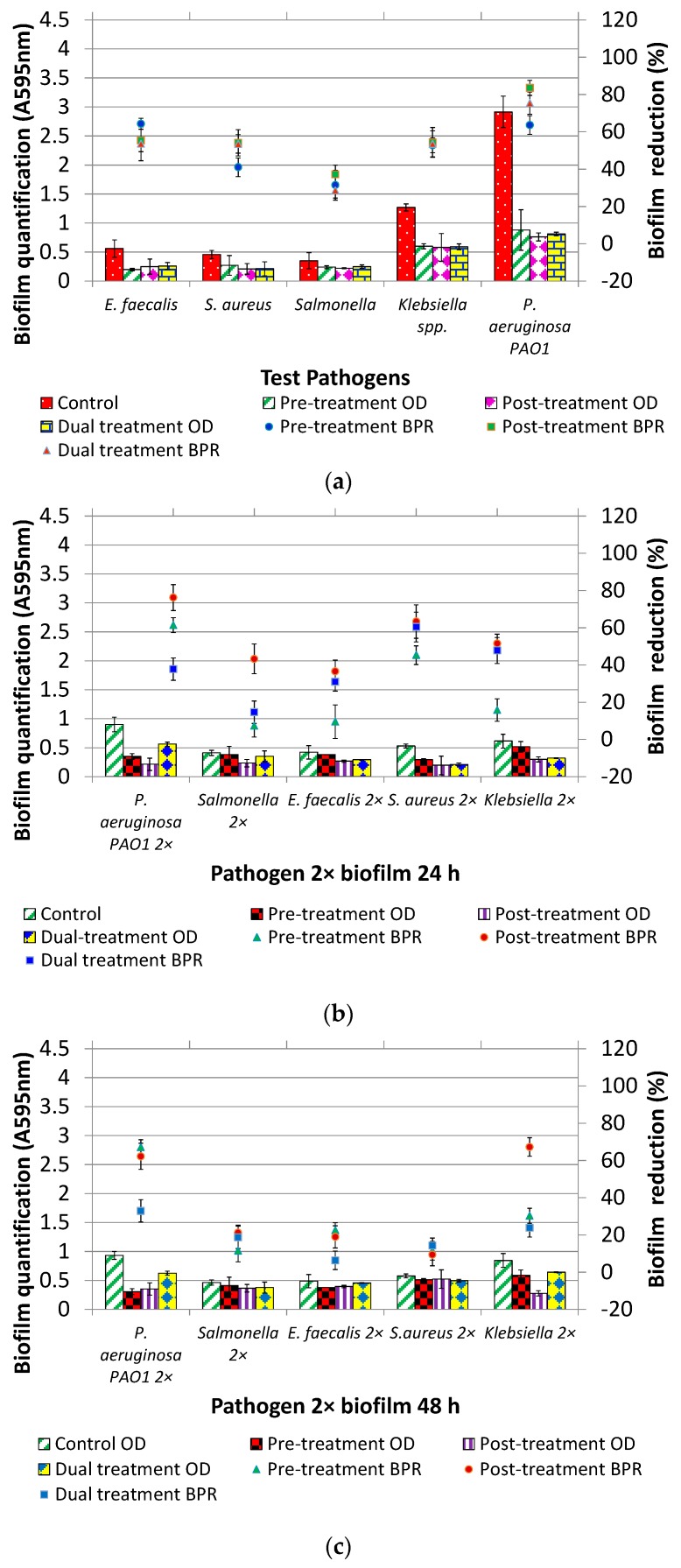
Effect of DNase I (without Mg^2+^) pre-treatment, post-treatment and dual treatment on biofilms formed by (**a**) test organism (individual); (**b**) test organism 2× mixed species biofilm formed for 24 h; (**c**) test organism 2× mixed species for biofilm formed for 48 h.

The effect of DNase I treatments (without Mg^2+^) on biofilm formation was evaluated in three sets of experiments: (Case-A) Individual pathogen: One test pathogen alone was used as inoculum to form biofilm for 24 h (Figure 3a); (Case-B) Pathogen 2× 24 h: mixed biofilm was formed for 24 h using all the pathogens at 1× inoculum level except one that was used at the 2× inoculum level. Hence, five different biofilms that were initiated with inoculum having one organism out of five at the 2× level and remaining at 1× (Figure 3b); (Case-C) Pathogen 2× 48 h: Similar to case b except the age of biofilm, which was 48 h (Figure 3c). 

The BPR as a result of three treatments (Pre, Post and Dual) seems to be similar for case-A, as evident by overlapping error bars in Figure 3a and statistical insignificant difference (*p* > 0.05). In reference to case-B, overall post-treatment was significantly better than pre-treatment (*p* < 0.05) but on par with dual-treatment (*p* > 0.05) except *P. aeruginosa* PAO1 2×, *Salmonella* Typhimurium 2×. Pre-treatment of *Salmonella* Typhimurium 2×, *E. faecalis* 2×, *Klebsiella* 2× with DNase I resulted in BPR of 7%, 9% and 15%, respectively. It is interesting to note that using pathogen at 2× inoculum level did not result in greater biofilm formation than when they were used at 1× inoculum level, not even in the case of *P. aeruginosa* PAO1 2×. However, at extended incubation time, control biofilm in case-C was significantly greater than that of case B (*p* < 0.05). In terms of BPR data, the efficacy of post-treatment was found to be reduced (*p* > 0.05) when pathogen 2× biofilm was grown for 48 h.

## 4. Discussion

Owing to several roles played by e-DNA in biofilm formation and strengthening of biofilm matrix, it has recently received much deserved attention by the research community. The current study involves pathogens like model biofilm forming organism *P. aeruginosa* PAO1 and other test organisms viz. *Klebsiella* spp., *S. aureus*, *E. faecalis* and *Salmonella* Typhimurium. These organisms either display biofilm mediated pathogenesis, or form enhanced biofilm in the presence of e-DNA, or are of relevance to the food industry [30]. The effect of DNase I on biofilm formation by test organisms was evaluated by pre-treatment, post-treatment and dual treatment. Optimization of treatments were done using *P. aeruginosa* PAO1 and the effect of the optimized treatments was evaluated on biofilm formation potential of individual and 24 and 48 h old mixed species biofilm.

The findings indicate that DNase I pretreatment (10 µg/mL) resulted in BPR of 68%. These findings can be corroborated with a previously published study that reported 40% reduction in biofilms when grown in the presence of 5 µg/mL DNase for 24 h [31]. Pretreatment of DNase at a concentration 5 µg/mL has been reported to reduce *E. coli* and *S. aureus* biofilm by 47–54% [32]. It can be concluded that DNase I concentration optimized in the current study for pre-treatment of biofilms is therefore comparable to the published literature.

Interestingly, we observed an inverse relation between the antibiofilm effect of DNase I pre-treatment and age of biofilm. Reduced vulnerability of the aged biofilm to DNase I indicates lower dependence of such biofilms on e-DNA. The mature biofilms might also scavenge the e-DNA in biofilm matrix to use it as a source of nutrition [8]. Moreover, the presence of DNase I throughout the process of biofilm formation (as during pre-treatment) may have propelled the biofilm to devise alternative strategies to compensate for the roles e-DNA plays [33]. The mature biofilm may still have e-DNA in the matrix but strengthening of the matrix by methods other than e-DNA in mature biofilms might render DNase ineffective. The results obtained in the current study are in complete agreement with a very recently published study wherein the antibiofilm effect of DNase was reported to be diminishing with the advancing age of biofilm [34]. Moreover, the efficacy of DNase is also dependent on availability of Mg^2+^ ion as discussed in detail in the following section.

After optimization of pre-treatment, post treatment was optimized at 10 µg/mL DNase I and contact duration of 15 min that resulted in 73–77% BPR. Most of the published studies have reported lower BPR at higher DNase concentrations and contact duration. A previously published study has reported 50% reduction in clinical *P. aeruginosa* biofilms when post-treated with glutathione and DNase (40U) [17]. Using DNase concentration almost 200 times higher, only 60% BPR could be achieved against preformed biofilms of *Acinetobacter baumannii* [10]. In another study with *Helicobacter pylori*, a very high concentration of DNase (1000 µg/mL) was used to achieve 50% BPR [35]. A previous report has documented 50% reduction in *L. monocytogenes* biofilm when pre-treated with DNase at concentration of 100 µg/mL and 75% reduction in case of post-treatment on 72 h old biofilm with contact duration of 24 h [22]. The basis for higher BPR achieved in this study further alludes that, in *P. aeruginosa* PAO1, e-DNA has a very crucial role to play in the process of biofilm formation [11,12].

Another valuable outcome of the current study is the synergistic effect of Mg^2+^ ions on efficacy of DNase I against *P. aeruginosa* PAO1 biofilms. Introduction of Mg^2+^ reduced the effective concentration of DNase I by 85% (reduction from 10 to 1.5 µg/mL) to achieve 80% and 75% BPR on polystyrene and polypropylene, respectively. We could not come across any study wherein introduction of Mg^2+^ has led to such a drastic increase in efficacy of DNase I. However, studies are available wherein introduction of Mg^2+^ has been reported to restore the antibiofilm effect of DNase I against *P. aeruginosa* biofilms [36]. Divalent ion, Mg^2+^ is a cofactor of DNase I and their addition seems to have improved the efficacy of the enzyme. These ions, however, have also been reported to reduce the efficacy of antibiotics [37,38]. Therefore, further studies are required for coming up with a strategy encompassing antimicrobials, DNase and Mg^2+^ ions for effective control of biofilms.

DNase I pre-treatment, post treatment and dual treatment (combination of pre-treatment and post treatment) on individual and mixed species biofilm revealed very interesting results. There was an insignificant difference in the effect of treatments on individual biofilms. The susceptibility of biofilms to DNase I was organism specific. These findings indicate that the biofilms vary with respect to their dependence on e-DNA for biofilm formation. To the best of the authors’ knowledge, there is a lack of reports wherein multiple pathogens have been compared in reference to antibiofilm effect of DNase I treatments (pre, post and dual); therefore, the findings could not be corroborated. 

The optimized DNase I treatments were tested against mixed species biofilms, which is a more accurate simulation of biofilms in real-life scenarios. The biofilms formed by test organisms individually were greater than that formed when the respective organism was dominant (2× inoculum) in mixed species. This observation can be attributed to the competitive and/or antagonistic interaction in the mixed species biofilm [39]. On the contrary, synergism amongst biofilm inhabitants has also been reported [30,40]. Overall, in the mixed species biofilm, DNase I was not as effective as against individual biofilms. The post treatments of mixed species biofilms grown for 24 h led to BPR in the range of 36–76%, which further declined to 13–53% with the ageing of biofilm for 48 h, except *Kelbsiella* 2× biofilms. Overall, mixed species biofilm is explored to lesser extent than individual biofilms and therefore we could not come across any study, wherein biofilms of more than two organisms were developed and treated with DNase I. The findings of the current investigation are slightly better than published studies on dual species biofilm of *Candida albicans* and *S. epidermidis* and *C. albicans* and *Streptococcus gordonii*, which have reported BPR of 35% and 25% [41,42]. Others have reported 45% and 80% reduction in viable cell count in dual species 48 h old biofilm formed by *L. monocytogenes*—*E. coli* and *L. monocytogenes*—*Pseudomonas fluorescens*, respectively, when post-treated with DNase (400 µg/mL, contact duration of 30 min) [43].

The prospect of using DNase I treatment as a part of clean-in-place regimes in the food industry are bolstered by the fact that it is heat sensitive and would be deactivated during heat treatments deployed in the food industry [44,45]. Moreover, if ingested along with the food items, acidic pH prevalent in stomach will degrade the enzyme [45]. DNase I based human therapeutics agents are also being developed for cystic fibrosis and rapid wound healing [46,47,48]. DNase I coating on polymethylmethacrylate biomaterial has been suggested for effective antibiotic delivery [49]. These reports indicate that time has ripened for the development of DNase based antibiofilm formulations for the food industry. 

## 5. Conclusions

In general, the findings of the current study indicate that post-treatment with DNase I was superior to pre-treatment and dual treatment even when applied to solo or mixed biofilms. In addition, DNase is effective to remove biofilms on various substrates used in the food industry like polypropylene and polystyrene. DNase itself is not an antimicrobial but can effectively sensitize the biofilm structure for antimicrobial. DNase can be considered for clean-in-place regimes in food industries in view of its efficacy in reducing biofilm formation or removing pre-existing biofilms. However, further research is required to understand the effect of DNase especially on mixed species biofilm in nature where conditions are not conducive for DNase.
